# *Ehrlichia chaffeensis* and Four *Anaplasma* Species With Veterinary and Public Health Significance Identified in Tibetan Sheep (*Ovis aries*) and Yaks (*Bos grunniens*) in Qinghai, China

**DOI:** 10.3389/fvets.2021.727166

**Published:** 2021-09-30

**Authors:** Ye Wang, Qingxun Zhang, Shuyi Han, Ying Li, Bo Wang, Guohui Yuan, Peiyang Zhang, Ziwen Yang, Heng Zhang, Yali Sun, Jiyong Chen, Xueqing Han, Hongxuan He

**Affiliations:** ^1^National Research Center for Wildlife Borne Diseases, Institute of Zoology, Chinese Academy of Sciences, Beijing, China; ^2^College of Agriculture, Ningxia University, Yinchuan, China; ^3^State Key Laboratory of Plateau Ecology and Agriculture, Qinghai University, Xining, China; ^4^College of Life Sciences, University of Chinese Academy of Sciences, Beijing, China; ^5^College of Animal Science, Anhui Science and Technology University, Chuzhou, China; ^6^Animal Disease Prevention and Control Center of Yushu, Yushu, China; ^7^Chinese Academy of Inspection and Quarantine, Beijing, China

**Keywords:** tick-borne disease, *Anaplasma capra*, *Ehrlichia chaffeensis*, Tibetan sheep, yak, Qinghai

## Abstract

Tick-borne diseases (TBDs) can cause serious economic losses and are very important to animal and public health. To date, research on TBDs has been limited in Qinghai-Tibet Plateau, China. This epidemiological investigation was conducted to evaluate the distribution and risk factors of *Anaplasma* spp. and *Ehrlichia chaffeensis* in livestock in Qinghai. A total of 566 blood samples, including 330 yaks (*Bos grunniens*) and 236 Tibetan sheep (*Ovis aries*) were screened. Results showed that *A. bovis* (33.3%, 110/330) and *A. phagocytophilum* (29.4%, 97/330) were most prevalent in yaks, followed by *A. ovis* (1.2%, 4/330), *A. capra* (0.6%, 2/330), and *E. chaffeensis* (0.6%, 2/330). While *A. ovis* (80.9%, 191/236) and *A. bovis* (5.1%, 12/236) infection was identified in Tibetan sheep. To our knowledge, it is the first time that *A. capra* and *E. chaffeensis* have been detected in yaks in China. Apart from that, we also found that co-infection of *A. bovis* and *A. phagocytophilum* is common in yaks (28.2%, 93/330). For triple co-infection, two yaks were infected with *A. bovis, A. phagocytophilum*, and *A. capra*, and two yaks were infected with *A. bovis, A. phagocytophilum*, and *E. chaffeensis*. Risk analysis shows that infection with *A. bovis, A. phagocytophilum*, and *A. ovis* was related to region and altitude. This study provides new data on the prevalence of *Anaplasma* spp. and *E. chaffeensis* in Qinghai, China, which may help to develop new strategies for active responding to these pathogens.

## Introduction

Anaplasmosis and ehrlichiosis are important diseases caused by tick-borne pathogens, which result in additional economic losses to livestock (1, 2). To date, seven *Anaplasma* species have been identified, including *A. bovis, A. phagocytophilum, A. centrale, A. platys, A. marginale, A. ovis*, and *A. capra* (3, 4). *A. bovis* parasitizes monocytes and macrophages of ruminants and small mammals (5). *A. phagocytophilum* infects neutrophils of humans and animals, and causing human granulocytic anaplasmosis (HGA), tick-borne fever in ruminants, and canine and equine granulocytic anaplasmosis (5). *A. centrale* and *A. marginale* mainly infect erythrocytes of cattle, while *A. ovis* primarily infect small ruminant animals such as sheep and goats. (6). *A. platys* mainly infect canine platelets and cause cyclic thrombocytopenia in dogs (6). *A. capra* is an emerging pathogen, which can infect ruminants and humans (7). In addition, as a member of the *Ehrlichia* family, *Ehrlichia chaffeensis* can cause human monocytic ehrlichiosis (HME) (8), and ehrlichiosis in animals (9).

Over the past several decades, the *Anaplasma* and *Ehrlichia* infections are very common in many countries (3, 10–12). *A*. *bovis* is mainly distributed in Africa, Asia, and South America, and cattle are considered the primary hosts (6). Similarly, *A. ovis* is the leading cause of anaplasmosis in small ruminants, which is widely distributed around the world (13). Recently, *A. phagocytophilum, A. capra*, and *E. chaffeensis* have received much attention for their potential threats to public health (7, 14). *A. phagocytophilum* has been detected in sheep, cattle, *Capreolus pygargus*, goats, and humans in different areas of China (15–18). *E. chaffeensis* infections are very common in the United States, with an annual rate of 4.46 cases/1,000,000 population (19). For *A. capra*, it was initially isolated from goats and humans in China (7). Subsequently, it was found in many countries (20, 21).

Qinghai is the source of the Yangtze River, the Yellow River, and the Lancang River, located in the northeast of Qinghai-Tibet Plateau and northwest of China with an average altitude of more than 3,000 meters. The complicated topographic features and changeable climate bless the region with advantageous conditions of rich natural resources. Tibetan sheep (*Ovis aries*) and yaks (*Bos grunniens*) are the main domestic animals in Qinghai and an important source of life and income for herders. Ixodid tick infestation in livestock is a common and severe problem, and more than 25 tick species in six genera have been reported in Qinghai (22, 23). However, information about tick-borne diseases (TBDs) in the region has been limited. Therefore, to better understand the situation of TBDs in Qinghai, China, a molecular epidemiologic study was conducted investigating exposure to *Anaplasma* spp. and *E. chaffeensis* in domestic animals across the area.

## Materials and Methods

### Blood Sample Collection of Yaks and Tibetan Sheep

**Figure 1:**
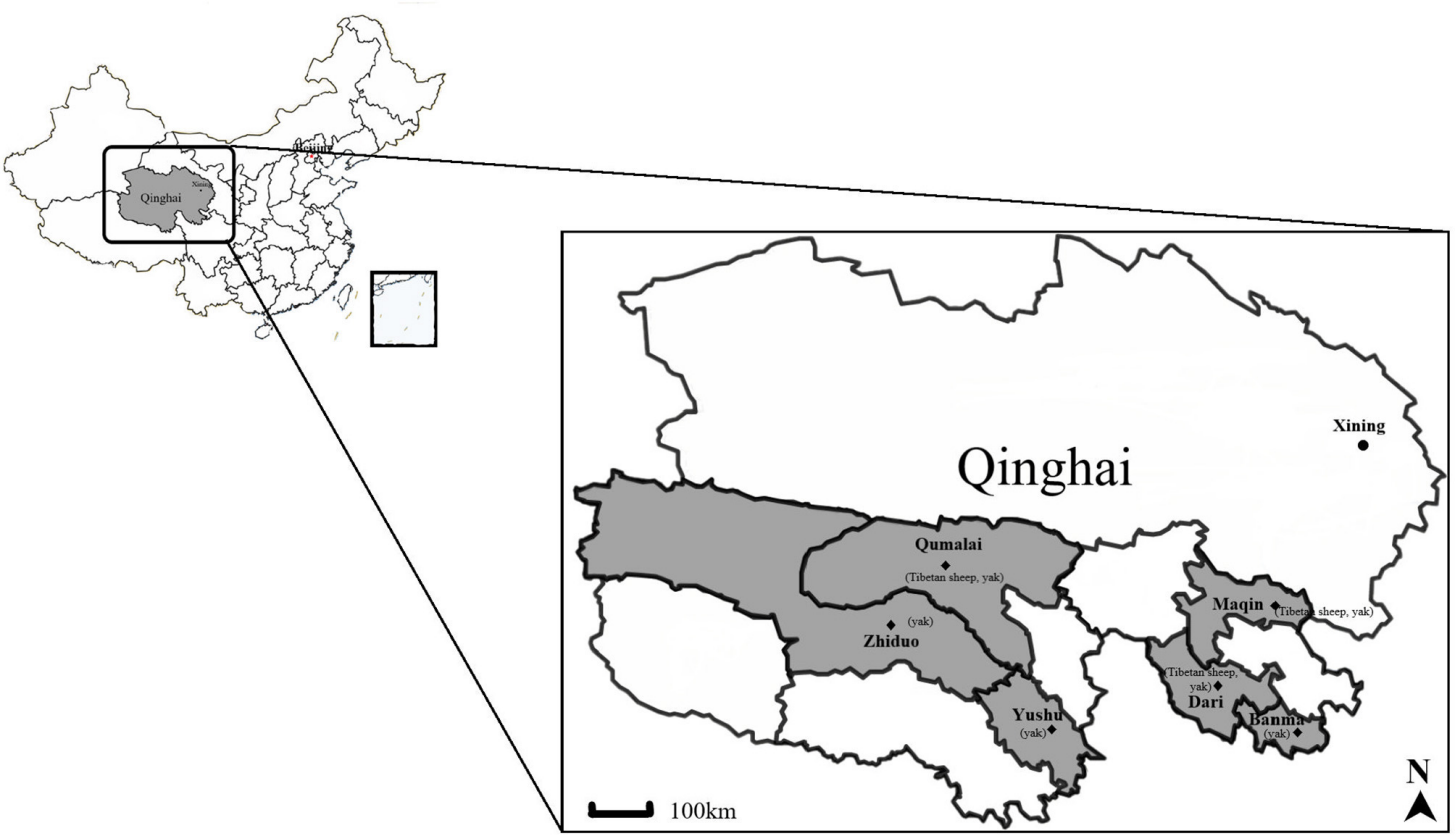
Map of the Qinghai Province, showing the sampling sites and animals included. The yak and Tibetan sheep samples were collected at six sites indicated by ♦. The figure was generated and modified using DITUHUI (https://g.dituhui.com).

A total of 566 blood samples of yaks (*n* = 330) and Tibetan sheep (*n* = 236) were collected using random sampling from six sampling sites in Maqin (35°2′38″N, 99°12′5″E; altitude 3,877 m), Dari (33°43′4″N, 99°38′2″E; altitude 4,130 m), and Banma (32°43′24″N, 100°42′41″E; altitude 3,864 m) of Guoluo Tibetan Autonomous Prefecture (GL), and Yushu (32°51′18″N, 96°48′57″E; altitude 4,317 m), Zhiduo (33°37′5″N, 95°58′51″E; altitude 4,177 m) and Qumalai (34°10′15″N, 95°49′57″E; altitude 4,279 m) of Yushu Tibetan Autonomous Prefecture (YS) during June 2020 in Qinghai, China (Figure 1). GL and YS are similar in altitude and climate, and both belong to the continental climate of the plateau. Except for about 400 Tibetan sheep in Maqin, the number of yaks and Tibetan sheep in other sampling sites is between 100 and 200. All animals adopt a free grazing system. Ticks and *Melophagus ovinus* and their bites can be seen in Tibetan sheep, while ticks are rarely found on yaks.

### Extraction and Quantification of DNA

According to the manufacturer's operation manual, genomic DNA was extracted from 200 uL whole blood samples by the TIANamp Genomic DNA kit (TIANGEN biotech, Beijing). The concentration of the extracted DNA was detected by NanoDrop 2,000 (Thermo Fisher Scientific, USA) and then stored at −20°C for pathogens detection.

### Detection of *Anaplasma* spp. and *E. chaffeensis*

Conventional PCR or nested PCR was used to screen for *Anaplasma* spp. and *E. chaffeensis* in extracted DNA. Nested PCRs were employed to detect *A. bovis, A. phagocytophilum, A. centrale, A. platys*, and *E. chaffeensis* based on *16S* rRNA gene. Conventional PCR based on the *msp4* genes was employed to detect *A. marginale* and *A. ovis*, while *16S* rRNA gene for detection of *A. capra*. PCR primers and cycling conditions used in this study, as shown in Table 1. The DNAs extracted from the whole blood of Tibetan sheep and yaks infected with *A. bovis, A. phagocytophilum, A. ovis, A. capra*, and *E. chaffeensis* that had been verified by sequencing, were used as a positive control for corresponding PCR reactions; double-distilled water was used as a negative control. The PCR products were detected by 1.5% agarose gel electrophoresis with M5 Hipure Next III Gelred (Mei5 Biotechnology Co., Ltd., Beijing, China) stained.

**Table 1:** Primers used in this study to detect *Anaplasma* spp. and *E. chaffeensis* in Tibetan sheep and yaks in Qinghai, China.

**Pathogens**	**Target gene**		**Primers (5^′^ → 3^′^)**	**Product (bp)**	**Annealing temperature (°C)**	**Reference**
*A. bovis*	*16S* rRNA	EE1	TCCTGGCTCAGAACGAACGCTGGCG	1,430	55	(24)
		EE2	AGTCACTGACCCAACCTTAAATGGCTG			
		AB1f	CTCGTAGCTTGCTATGAGAAC	551	55	(12)
		AB1r	TCTCCCGGACTCCAGTCTG			
*A. phagocytophilum*	*16S* rRNA	EE1	TCCTGGCTCAGAACGAACGCTGGCG	1,430	55	(24)
		EE2	AGTCACTGACCCAACCTTAAATGGCTG			
		SP2f	GCTGAATGTGGGGATAATTTAT	641	55	(12)
		SP2r	ATGGCTGCTTCCTTTCGGTTA			
*A. centrale*	*16S* rRNA	EE1	TCCTGGCTCAGAACGAACGCTGGCG	1,430	55	(24)
		EE2	AGTCACTGACCCAACCTTAAATGGCTG			
		AC1f	CTGCTTTTAATACTGCAGGACTA	426	60	(17)
		AC1r	ATGCAGCACCTGTGTGAGGT			
*A. platys*	*16S* rRNA	EE1	TCCTGGCTCAGAACGAACGCTGGCG	1,430	55	(24)
		EE2	AGTCACTGACCCAACCTTAAATGGCTG			
		Apf	TCCTGGCTCAGAACGAACGCTGGCGGC	506	60	(17)
		APr	AGTCACTGACCCAACCTTAAATGGCTG			
*A. marginale/ A. ovis*	*msp4*	MSP45	GGGAGCTCCTATGAATTACAGAGAATTGTTTAC	870	60	(25)
		MSP43	CCGGATCCTTAGCTGAACAGGAATCTTGC			
*A. capra*	*16S* rRNA	Capra-F	GCAAGTCGAACGGACCAAATCTGT	1,261	58	(26)
		Capra-R	CCACGATTACTAGCGATTCCGACTTC			
*E. chaffeensis*	*16S* rRNA	ECB	CGTATTACCGCGGCTGCTGGCA	450	60	(27)
		ECC	AGAACGAACGCTGGCGGCAAGCC			
		HE1	CAATTGCTTATAACCTTTTGGTTATAAAT	3,90	55	(27)
		HE3	TATAGGTACCGTCATTATCTTCCCTAT			

### Sequencing and Phylogenetic Analysis

PCR products of all positive samples for *Anaplasma* spp. and *E. chaffeensis* randomly selected from each sampling site were sequenced by BGI (Beijing, China). The sequence obtained by BGI sequencing was submitted to NCBI for BLASTn search (https://blast.ncbi.nlm.nih.gov/Blast.cgi), and sequence alignment and analysis. The representative nucleotide sequences of this study have been deposited in the GenBank database. Phylogenetic trees were constructed using the neighbor-joining method executed with the p-distance model in MEGA X. Bootstrap values were assessed with 1,000 bootstrap replicates (28, 29).

### Statistical Analysis

The data were grouped into four variables according to animal species, gender, sampling sites, and the altitude of sampling sites. The chi-square test was used to calculate the difference of infection rate in SPSS 25.0 software in each group. When *p* < 0.05, the difference was significant.

## Results

### Prevalence of *Anaplasma* spp. and *E. chaffeensis* in Tibetan Sheep and Yaks

This study identified four *Anaplasma* species and *E. chaffeensis* from Tibetan sheep and yaks (Table 2). Of the 566 samples tested, 50% (283/566) were positive for at least one pathogen. The infection rates of *A. bovis* and *A. ovis* were 33.3% and 1.2% in yaks, 5.1% and 80.9% in Tibetan sheep. The infection rates of *A. phagocytophilum, A. capra*, and *E. chaffeensis* were 29.4%, 0.6%, and 0.6% in yaks, respectively. This is the first time that *A. capra* and *E. chaffeensis* have been detected in yaks in China. Interestingly, we noticed *A. ovis* infection in yaks and *A. bovis* in Tibetan sheep. The most common co-infection was *A. bovis* and *A. phagocytophilum*, with an infection rate of 28.2% (93/330) in yaks. For co-infection with three pathogens, the infection rate of *A. bovis, A. phagocytophilum*, and *A. capra* was 0.6% (2/330), and the infection rate of *A. bovis, A. phagocytophilum*, and *E. chaffeensis* was 0.6% (2/330) (Table 2). No co-infections by two or more pathogens were detected in Tibetan sheep.

**Table 2:** The prevalence of *Anaplasma* spp. and *E. chaffeensis* in Tibetan sheep and yaks in Qinghai, China.

		GL^*^				YS^*^			
**Species**	**Pathogens**	**No. infected/(%)**				**No. infected/(%)**			
		**Maqin**	**Dari**	**Banma**	**Total**	**Yushu**	**Qumalai**	**Zhiduo**	**Total**
Yak	No. tested	95	35	84	**214**	56	30	30	**116**
	*A. bovis*	1 (1.1)	0	84 (100)	**85 (39.7)**	21 (37.5)	0	4 (13.3)	**25 (21.6)**
	A. phago^*^	0	0	74 (88.1)	**74 (34.6)**	19 (33.9)	1 (3.3)	3 (10)	**23 (19.8)**
	*A. ovis*	0	0	0	**0**	0	4 (13.3)	0	**4 (3.5)**
	*A. capra*	0	0	2 (2.4)	**2 (0.9)**	0	0	0	**0**
	*E. chaffeensis*	0	0	1 (1.2)	**1 (0.5)**	1 (1.8)	0	0	**1 (0.9)**
	*A. bovis + A. phago*	0	0	74 (88.1)	**74 (34.6)**	16 (28.6)	0	3 (10)	**19 (16.4)**
	*A. bovis + A. phago + A. capra*	0	0	2 (2.4)	**2 (0.9)**	0	0	0	**0**
	*A. bovis + A. phago + E. chaffeensis*	0	0	1 (1.2)	**1 (0.5)**	1 (1.8)	0	0	**1 (0.9)**
Tibetan sheep	No. tested	143	51	0	**194**	0	42	0	**42**
	*A. bovis*	12 (8.4)	0	0	**12 (61.9)**	0	0	0	**0**
	*A. ovis*	109 (76.2)	48 (94.1)	0	**157 (80.9)**	0	34 (81)	0	**34 (81)**

* *A. phago = A. phagocytophilum, GL: Guoluo Tibetan Autonomous Prefecture, YS: Yushu Tibetan Autonomous Prefecture*.

### Sequencing and Phylogenetic Analysis

**Figure 2:**
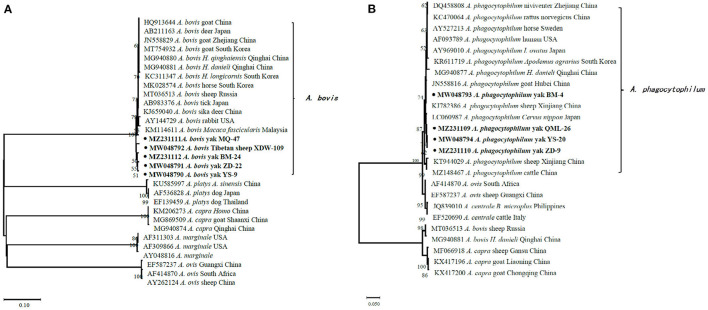
Phylogenetic trees were constructed based on partial sequences of the *16S* rRNA genes of *A. bovis* **(A)** and *A. phagocytophilum* **(B)**. Phylogenetic trees were con-structed by the neighbor-joining method with the p-distance model using the MEGA X software, and the bootstrap test was assessed with 1,000 replicates. The species identified in this study are indicated by • and highlighted in bold.

**Figure 3:**
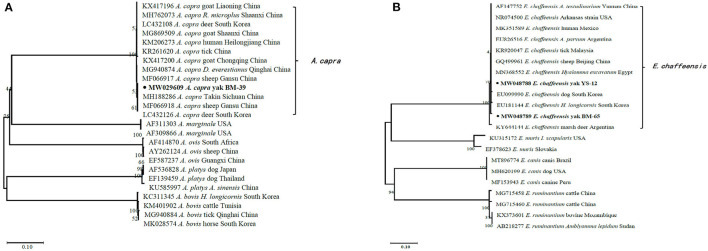
Phylogenetic trees were constructed based on partial sequences of the 16S rRNA genes of *A. capra* **(A)** and *E. chaffeensis* **(B)**. Phylogenetic trees were constructed by the neighbor-joining method with the p-distance model using the MEGA X software, and the bootstrap test was assessed with 1,000 replicates. The species identified in this study are indicated by • and highlighted in bold.

**Figure 4:**
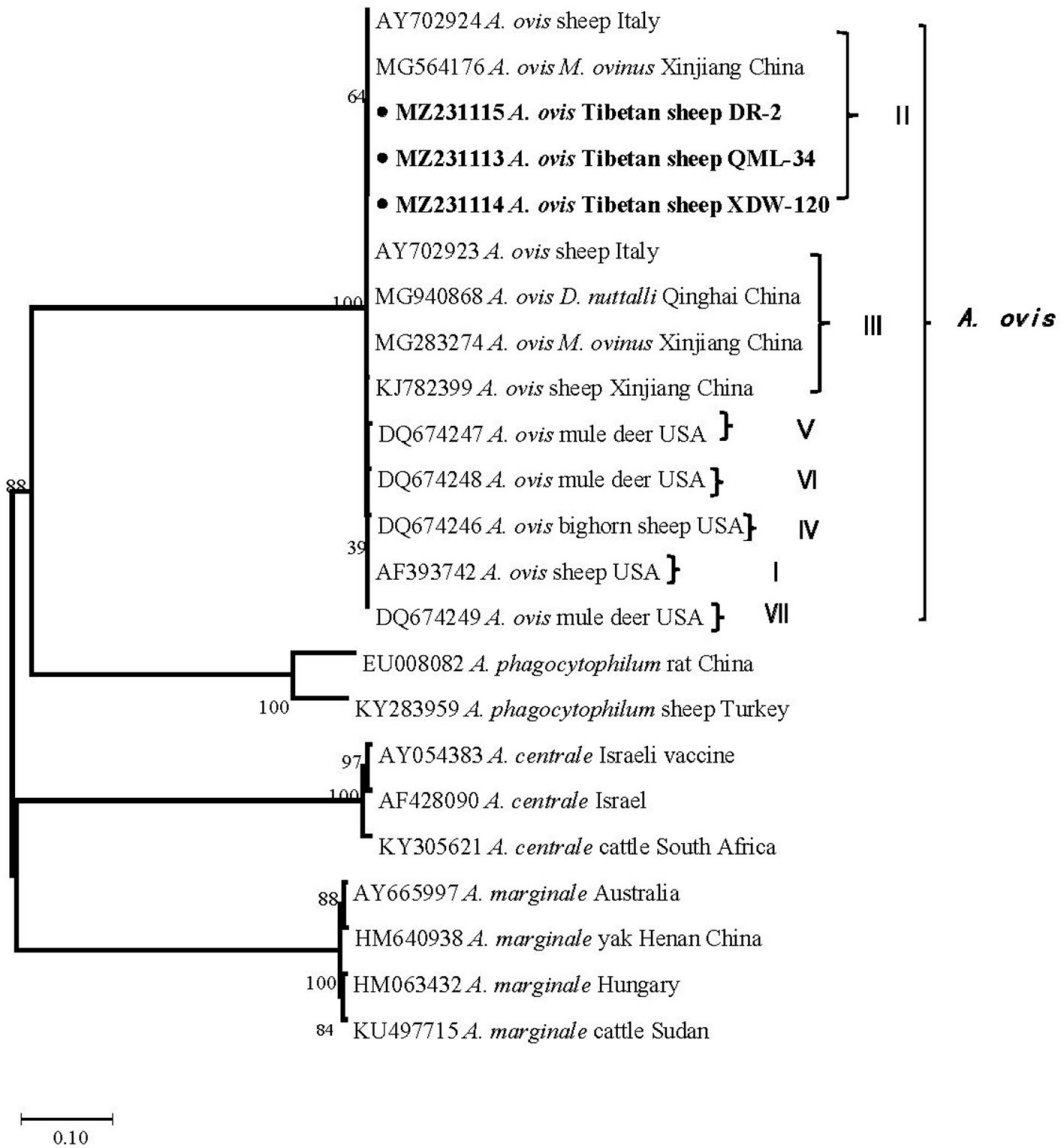
Phylogenetic trees were constructed based on the msp4 genes of *A. ovis*. Phylogenetic trees were constructed by the neighbor-joining method with the p-distance model using the MEGA X software, and the bootstrap test was assessed with 1,000 replicates. The species identified in this study are indicated by • and highlighted in bold.

In the current study, 15 representative sequences were obtained and submitted to GenBank (Table 3). We compared and analyzed the partial *16S* rRNA gene sequences of *A. bovis, A. phagocytophilum, A. capra*, and *E. chaffeensis* obtained from blood samples of Tibetan sheep and yaks. BLASTn analysis of the *16S* rRNA gene showed that the *Anaplasma* spp. obtained in this study had 99.04–100% identities to either of *A. bovis, A. phagocytophilum, A. capra*, and *E. chaffeensis* sequences, respectively. The *E. chaffeensis* sequences (MW048788, MW048789) from yaks were 99.44–100% identical to *E. chaffeensis* isolated from goats (KX505292) in China. Phylogenetic analysis of *16S* rRNA gene sequences confirmed *A. bovis, A. phagocytophilum, A. capra*, and *E. chaffeensis* in this study (Figures 2A,B, 3A,B). Additionally, we analyzed the *msp4* genomic region of three *A. ovis* (MZ231113-MZ231115) obtained in this study. The results showed that the three sequences were consistent with the homology of the Iranian *A. ovis* (MH790273). *A. ovis* were classified as *A. ovis msp4* Genotypes II based on T^366^C^470^ (25). Phylogenetic analysis of *msp4* gene sequences confirmed the identity of *A. ovis* in this study (Figure 4).

**Table 3:** The DNA sequences submitted to the gene bank in this study.

**Obtained sequences**					**Reference sequences from GenBank**	
**Pathogen**	**Host**	**Target gene**	**Accession number**	**Length (bp)**	**Identity (%)**	**Accession number (host, country)**
*A. bovis*	yak	*16S* rRNA	MW048790	516	99.61	MT036513 (sheep, Russia)
	yak	*16S* rRNA	MW048791	525	99.04	MT036513 (sheep, Russia)
	Tibetan sheep	*16S rRNA*	MW048792	524	99.42	MT036513 (sheep, Russia)
	yak	*16S rRNA*	MZ231111	524	99.61	MT036513 (sheep, Russia)
	yak	*16S rRNA*	MZ231112	525	99.81	MN213735 (giraffe, Pakistan)
*A. phago*	yak	*16S rRNA*	MW048793	620	99.34	MW142385 (*M. ovinus*, China)
	yak	*16S rRNA*	MW048794	617	99.67	MW142385 (*M. ovinus*, China)
	yak	*16S rRNA*	MZ231109	618	99.83	MW142385 (*M. ovinus*, China)
	yak	*16S rRNA*	MZ231110	617	99.67	MW142385 (*M. ovinus*, China)
*A. capra*	yak	*16S rRNA*	MW577114	1106	100	MF066918 (sheep, Gansu)
*A. ovis*	Tibetan sheep	*msp4*	MZ231113	826	100	MH790273 (sheep,Iran)
	Tibetan sheep	*msp4*	MZ231114	824	100	MH790273 (sheep,Iran)
	Tibetan sheep	*msp4*	MZ231115	824	100	MH790273 (sheep,Iran)
*E. chaffeensis*	yak	*16S* rRNA	MW048788	360	100	KX505292 (goat, China)
	yak	*16S* rRNA	MW048789	362	99.44	KX505292 (goat, China)

### Risk Factors of Tibetan Sheep and Yaks Infected With *Anaplasma* spp. and *E. chaffeensis*

These factors include animal species, gender, sampling sites, and altitude of sampling sites, which were used as variables for statistical analysis of the infection patterns of *Anaplasma* spp. and *E. chaffeensis*. The results indicate that the prevalence of *Anaplasma* spp. and *E. chaffeensis* in female animals was similar to that of male animals (*P* >0.05). The infection rates of *A. bovis, A. phagocytophilum*, and *A. ovis* in yaks in GL and YS were 39.7 and 21.6% (*P* = 0.001), 34.6 and 19.8% (*P* = 0.006), 0 and 3.5% (*P* = 0.005), respectively. In addition, the infection rate of *A. bovis* and *A. phagocytophilum* below 4,000 m was significantly higher than those above 4,000 m (*P* = 0.000). In Tibetan sheep, the infection rate of *A. ovis* above 4,000 m was higher than that below 4,000 m (*P* = 0.022) (Table 4).

**Table 4:** Patterns of *Anaplssasma* spp. and *E. chaffeensis* prevalence in the yaks and Tibetan sheep, grouped by animal species, gender, sampling sites, and the altitude of sampling sites.

		**Yak**										**Tibetan sheep**			
	**Parameter**	**No. infected/(%)**										**No. infected/(%)**			
		***A. bovis***	***p*-value**	***A. phago***	***p*-value**	***A. ovis***	***p*-value**	***A. capra***	***p*-value**	***E. chaffeensis***	***p*-value**	***A. bovis***	***p*-value**	***A. ovis***	***p*-value**
Gender	Female	69 (33.8)	0.810	58 (28.4)	0.625	3 (1.5)	0.585	0	0.071	2 (1)	0.265	10 (4.8)	0.597	168 (80.8)	0.862
	Male	41 (32.5)		39 (31)		1 (0.8)		2 (1.6)		0		2 (7.1)		23 (82.1)	
Sampling sites	GL	85 (39.7)	0.001^*^	74 (34.6)	**0.006**	0	**0.005**	2 (0.9)	0.296	1 (0.5)	0.659	12 (61.9)	0.098	157 (80.9)	0.997
	YS	25 (21.6)		23 (19.8)		4 (3.5)		0		1 (0.9)		0		34 (81)	
Altitude	3,500–4,000 m	85 (47.5)	**0.000**	74 (41.3)	**0.000**	0	**0.028**	2 (1.1)	0.193	1 (0.6)	0.904	12 (8.4)	**0.04**	109 (76.2)	**0.022**
	>4000 m	25 (16.6)		23 (15.2)		4 (2.7)		0		1 (0.7)		0		82 (88.2)	

* *, Bold indicates significant difference*.

## Discussion

In the present study, *Anaplasma* spp. and *E. chaffeensis* were investigated in domestic animals in Qinghai, China. Four *Anaplasma* species (*A. bovis, A. phagocytophilum, A. ovis*, and *A. capra*) and *E. chaffeensis* were identified in Tibetan sheep and yaks. Among them, *E. chaffeensis* and *A. capra* were detected in yaks for the first time in China.

The genus *Anaplasma* are widely distributed in domestic animals, wild animals, ticks, and other vectors (23, 30–32). This study found relatively high *A. ovis* infection rates of 76.2, 94.1, and 81.3% in Tibetan sheep in three sampling sites, Maqin, Dari, and Qumalai, respectively, which is higher than in sheep in Xinjiang (40.5%) (16) and Gansu (5.7%) (33), and Tibetan sheep in northeast Qinghai (58%) (34). An explanation for higher infection rates of *A. ovis* in this area could be the bites of ticks and other arthropods. Ticks and *M. ovinus* were found in Tibetan sheep in Maqin and Dari, and data on that *M. ovinus* carried *A. ovis* has been reported in our previous study (31). In addition, we carried out the comparative analysis and phylogenetic analysis of the *msp4* gene sequence of *A. ovis* (25). The results showed that the *A. ovis* strains isolated from Tibetan sheep were identical to those isolated in *M. ovinus* in our previous study (31). Whereas, the *A. ovis* isolated from *Dermacentor nuttalli* in Qinghai by Han et al. (23) belongs to genotypes III, which is in the same clade as those obtained from sheep in Italy (Figure 4) (25). Genotypes II and III were also isolated from *M. ovinus* in Xinjiang by Zhao et al. (35). Remarkably, an *A. ovis* variant was reported in humans (36), indicating that this agent has zoonotic potential. Taken together, there are two *A. ovis* genotypes prevalent in domestic animals in northwest China, and arthropods (including *M. ovinus* and ticks) may be the main vectors of *A. ovis*.

*A. phagocytophilum* and *A. bovis* are frequently detected in ruminants around the world. This study confirms that both *A. phagocytophilum* and *A. bovis* can infect yaks. The infection rate of *A. phagocytophilum* in yaks (29.4%) in this study was higher than that reported in sheep (9.9%), dairy cattle (12%), and white yaks (5.3%) in other areas of China (1, 13, 37), and lower than that in *C. pygargus* (33.3%) from Heilongjiang China (17). Since the first case of HGA, caused by *A. phagocytophilum*, was reported in Anhui, China (38), HGA has been reported in the USA, Europe, Africa, and Asia (11, 39, 40). For *A. bovis*, the infection rate in yaks (33.3%) was higher than that in cattle (4.8%) and white yaks (6.2%) from China (16, 37), cattle (1.0%) from South Korea (20). Recent studies have shown that climate, altitude, longitude, latitude, season, tick bites, contact with wild animals, and feeding methods are important factors affecting *Anaplasma* infection (41). Previous reports have shown that *Haemaphysalis qinghaiensis, Dermacentor abaensis, D. nuttalli*, and *Dermacentor silvarum* are common ectoparasites among grazing livestock in high altitude areas (2,800 to 4,300 m), and the risk of tick bites with *Anaplasma* spp. was related to altitude and tick species (23). Our results also showed that the risk of infection with *Anaplasma* spp. in Tibetan sheep and yaks is mainly related to altitude and sampling sites. Furthermore, all animals in this study adopted a free grazing system, which increased the risk of domestic animals being exposed to ticks.

*A. capra* is a novel *Anaplasma* species that emerged in recent years. The novel species was first found in goats and then in sheep (30), *C. pygargus* (17), dogs (42), and ticks (23) in China. In addition, *A. capra* has also been detected in goats, cattle, and *Hydropotes inermis argyropus* in South Korea (32, 43), cattle in Malaysia (10), and *Cervus elaphus* and *Rucervus duvaucelii* in France (21). In 2015, it was isolated from the blood samples of patients with a history of tick bites in northeastern China (7). Subsequently, Peng et al. (44) confirmed the ability of *A. capra* to infect human erythrocytes, HL-60 and TF-1, and further confirmed its zoonotic characteristics. In this study, we detected *A. capra* DNA in yaks in China for the first time. In Qinghai, *H. qinghaiensis* is the most dominant tick species infected with *A. capra*, followed by *D. abaensis* and *D. nuttalli* (23). The above evidence suggests that *A. capra* is widely distributed and could infect a wide range of hosts.

*Ehrlichia* species include *E. chaffeensis, E. canis, E. ewingii, E. equi, E. muris*, and *E. ruminantium*. These species have been detected in many ticks in China, for instance, *Amblyomma testudinarium, Haemaphysalis yeni, Haemaphysalis longicornis, Ixodes sinensis, D. silvarum, Rhipicephalus sanguineus*, and *Rhipicephalus microplus* (45–48). In previous studies, *E. cani*s infection was detected in *Cervus nippon* in Gansu (49), and high infection rates of *E. cani*s and *E. chaffeensis* were reported in dogs, cattle, sheep, goats, donkeys, and humans in Xinjiang (9, 18, 50). *Ehrlichia* species were also detected in birds and small mammals in other parts of China (51, 52). In the current study, the prevalence rate of *E. chaffeensis* was 0.61%. We present the first report of *Ehrlichia* infection caused by *E. chaffeensis* in yaks in China. However, it is unclear which ticks are responsible for the pathogen. Therefore, further study is needed to determine the vector or reservoir host for this pathogen.

Moreover, mixed-infection is also an important issue that would need to be considered in livestock. The present study results illustrate that mixed infection of *A. phagocytophilum* and *A. bovis* are very common in yaks in Qinghai. Co-infection involving three *Anaplasma* species of *A. bovis, A. phagocytophilum*, and *A. capra* was also observed in two yaks in this study. In addition, we found that two yaks were co-infected with *A. bovis, A. phagocytophilum* and *E. chaffeensis*. Currently, *A. phagocytophilum, A. capra*, and *E. chaffeensis* have been recognized as causative agents of human infection. Mixed-infection of tick-borne pathogens has also been observed in animals in other countries and regions (1, 30, 34, 53). Above all, co-infection of tick-borne pathogens emphasizes the need for differential diagnosis of these pathogens in animal hosts and humans to improve the prevention and control of TBDs.

Notably, all pathogens were detected from apparently healthy animals in this study, consistent with other studies (54–56). This indicates that the appearance of clinical symptoms is mainly dependent on the pathogenicity of these pathogens strains and the breed or species of the infected animals (54). Alternatively, these animals have previously been infected with these pathogens and developed immunity against these pathogens (56). Further research is necessary to confirm these speculations.

In conclusion, we investigated the epidemic situation of the TBDs in yaks and Tibetan sheep in Qinghai province, China, and confirmed that Tibetan sheep and yaks could be infected with *A. bovis, A. phagocytophilum, A. ovis, A. capra*, and *E. chaffeensis*. This is the first report of *A. capra* and *E. chaffeensis* infection in yaks in China. These pathogens could pose a significant threat to livestock and human health. Thus, future studies should focus more on systematically assessing these pathogens' threats to veterinary and public health.

## Data Availability Statement

The datasets presented in this study can be found in online repositories. The names of the repository/repositories and accession number(s) can be found in the article/supplementary material.

## Ethics Statement

The Animal Ethics Committee of the Institute of Zoology, Chinese Academy of Sciences approved the procedures of collecting blood samples from Tibetan sheep and yaks, and obtained the livestock owner's consent. Written informed consent for participation was not obtained from the owners because all the samples in this study were collected by local veterinarians during the daily epidemic surveillance.

## Author Contributions

YW: investigation, conceptualization, methodology, data curation, visualization, writing—original draft, and writing—review & editing. QZ: investigation, methodology, visualization, writing—original draft, and writing—review & editing. YL: investigation, methodology, data curation, and funding acquisition. SH: investigation, methodology, and writing—original draft. BW, GY, PZ, ZY, and HZ: investigation and methodology. YS, XH, and JC: investigation. HH: investigation, visualization, supervision, validation, writing—review & editing, and funding acquisition. All authors contributed to the article and approved the submitted version.

## Funding

This work was supported by the Regular Assistance Project of the International Department of the Ministry of Science and Technology of China (KY201904013), the Chinese Academy of Sciences (CZBZX-1), National Forestry, and Grassland Administration, China.

## Conflict of Interest

The authors declare that the research was conducted in the absence of any commercial or financial relationships that could be construed as a potential conflict of interest.

## Publisher's Note

All claims expressed in this article are solely those of the authors and do not necessarily represent those of their affiliated organizations, or those of the publisher, the editors and the reviewers. Any product that may be evaluated in this article, or claim that may be made by its manufacturer, is not guaranteed or endorsed by the publisher.
